# Surgical Cases

**Published:** 1846-04-01

**Authors:** B. F. Hastings


### Surgical Cases.

Communicated for the Buffalo Medical Journal, by B. F. HASTINGS, M. D.

NILES, MICHIGAN.

DR. FLINT:—I see and read your Journal in these far off regions, and while reading I have thought you might be glad to hear how we practice who live remote from the fountains of Medical Science.

*Fracture of Tibia and Fibula.*—Mr. C., aged 35, fell from a warehouse, fifty feet. I saw patient fifteen minutes after, found fractures of Tibia and Fibula oblique; applied Scultetus, bandage (I prefer it to the properly called 18 tailed) and Desault's straight splint, using moderate extension. At the end of five days, substituted double inclined plane for straight splint, with evident relief to patient. Five weeks from time of accident he walked to my office—very slight displacement at seat of fracture in tibia, but who does better in oblique fractures of large bones?

*Compound comminuted Fracture of Tibia and Fibula.*—David B. aged 7—Caught his right leg in the "horse-power" of a thrashing machine, producing an extensive laceration of the gastrocnemii, &c., with compound comminuted fractures of tibia and fibula. Drs. Richardson and myself reduced the bones, and closed the wound with sutures, hoping to reduce it by a prompt adhesion to a simple fracture—limb placed in a box and carefully supported.

The limb is saved; but such a limb by the army and by many "hospital" surgeons would have been sacrificed at once. A true surgeon, we believe should be known by the number of limbs he saves, and not by the number he cuts off. To Dr. Richardson, who subsequent to the first dressing alone attended the patient, set down the credit.

*Fracture of Phalanx of Index Finger.*—Union perfect, and of course it ought to be.

*Partial luxation of Tibia backwark.*—H. R., aged 28—was dragged by a horse with his foot entangled in a stirup. Dr. Manning and myself were called immediately, and while prtient was suffering under great prostration, the reduction was made with ease by pulling in a straight line nearly:—I would have thanked any one to have told me before how easily such a grave dislocation might be reduced, it would have saved me several cold chills; remember, however, it was *partial.*

Thus you have a small digest, or a sort of current diary of my "sylvan" surgery. I think it may be worth as much as some things which are thought to be worth more; if you think so, well—print it: if not, just as well—burn it.

Yours, truly,

B. F. HASTINGS.

*Niles, St. Josephs Co., Michigan.*

